# The Streaming Potential of Fluid through a Microchannel with Modulated Charged Surfaces

**DOI:** 10.3390/mi13010066

**Published:** 2021-12-30

**Authors:** Xinyue Bian, Fengqin Li, Yongjun Jian

**Affiliations:** School of Mathematical Science, Inner Mongolia University, Hohhot 010021, China; 32036046@mail.imu.edu.cn (X.B.); jianyj@imu.edu.cn (Y.J.)

**Keywords:** modulated charged potential, electric double layer (EDL), streaming potential, microchannel

## Abstract

In this paper, the effects of asymmetrically modulated charged surfaces on streaming potential, velocity field and flow rate are investigated under the axial pressure gradient and vertical magnetic field. In a parallel-plate microchannel, modulated charged potentials on the walls are depicted by the cosine function. The flow of incompressible Newtonian fluid is two-dimensional due to the modulated charged surfaces. Considering the Debye–Hückel approximation, the Poisson–Boltzmann (PB) equation and the modified Navier-Stokes (N-S) equation are established. The analytical solutions of the potential and velocities (*u* and *v*) are obtained by means of the superposition principle and stream function. The unknown streaming potential is determined by the condition that the net ionic current is zero. Finally, the influences of pertinent dimensionless parameters (modulated potential parameters, Hartmann number and slip length) on the flow field, streaming potential, velocity field and flow rate are discussed graphically. During the flow process and under the impact of the charge-modulated potentials, the velocity profiles present an oscillating characteristic, and vortexes are generated. The results show that the charge-modulated potentials are beneficial for the enhancement of the streaming potential, velocity and flow rate, which also facilitate the mixing of fluids. Meanwhile, the flow rate can be controlled through the use of a low-amplitude magnetic field.

## 1. Introduction

In recent years, microfluidics systems have begun to receive more attention from scholars due to the lab-on-a-chip concept [1,2,3,4,5]. This is a new research direction that involves biomedicine, chemistry and fluid physics [6,7,8,9]. The driving forms of this new type of technology include the pressure gradient, electric field, magnetic field and surface tension. Traditionally, single pressure driving is the predominant driving form. In order to achieve better fluid control in a microchannel, a method that combines the pressure, electric field, magnetic field and other driving sources as the driving form of microchannel has become widely used [10,11,12,13,14]. In recent years, many studies have tended to use magnetic fields to control the flow rate in microchannels [15,16]. In reality, due to the lack of precision in the actual manufacturing process, it is possible for defects to be present in the walls of microchannels. Consequently, it is necessary to take the modulated charged surfaces into consideration.

During the research process, the free ions that are in the solution and the charged ions that are on the surfaces are redistributed by attraction and repulsion to produce an electric double layer (EDL). Because there is an ionic equilibrium in the EDL, we can use the Poisson–Boltzmann (PB) equation to relate the ion concentration to the potential. However, the idealized state of uniform electric potential is often considered in previous studies. In actual production and application, we cannot achieve uniform channel walls for microdevices. However, uniform electric potentials on the walls will ignore the change of vertical ion concentration. In this sense, the modulated charged potentials will be considered in this study. The modulated charged surfaces are used in microchannels to replace the nonsmooth surfaces that are caused due to the defects that occur during the manufacturing process. The presence of axially modulated surface charge leads to an axial velocity gradient, which results in a transverse component of velocity in order to satisfy continuity equation. Therefore, the vertical ion concentration will change with different vertical distance. The vertical velocity component is generated because of the modulated charged potentials, resulting in more complicated phenomena taking place in the microchannel. The study of Ghosal [17] showed that the applied pressure and the electric potential both had a linear relationship with volume flux in the microchannel. This conclusion is used to study the dispersion in the microchannel. Wei [18] theoretically studied the influence of charge modulated on EO flow by imposing shear flow. They further explored the more complex flow that was generated by time modulation. The work of Ghosh and Chakraborty [19] reveals an optimal pattern frequency that can be used to achieve the most efficient microfluidic mixing within constraints. Bandopadhyay and Ghosh et al. [20] studied the EO flow of viscoelastic fluid. Studies have shown that the distribution of the flow field can be changed by changing the charge pattern. Datta and Choudhary [21] studied the influence of slip boundary conditions on the electroosmotic (EO) flow under the wall potential changes periodically in the nanochannel. Ghosh and Chakraborty [22] studied the induced streaming electric field in the presence of patterned surface wettability and modulated surface charges. Ng and Qi [23] established a model of power-law fluid in narrow channels. They found that the walls surface after modulation would cause nonlinear behavior of non-Newtonian fluid flow by changing channel height and wall potential. Mandal and Ghosh et al. [24] established an asymmetric wall potential mode. In the presence of axially modulated surface charges, they analyzed the EO flow of two superimposed fluids and found that the flow lines were deformed to different degrees. Ghosh and Chakraborty [25] investigated how to enhance microfluidic mixing by exploiting electrokinetic transport of viscoelastic fluids over charge modulated surfaces. Qi and Ng [26] studied the influence of non-uniform walls modulation on the flow by considering the mechanism of a two-fluid EO system. Jimenez and Escandón et al. [27] studied the electro-osmotic flow considering the viscoelectric and steric effects for mixing applications.

The relative movement of an electrolyte solution produces an electrokinetic phenomenon. Common electrokinetic phenomena are electro-osmosis and streaming potential. Although there is no applied electric field, pressure is the main force that drives fluid flow. Due to the existence of EDL, the counterions move towards the downstream and accumulate at the end of microchannel. Finally, an electrokinetic potential, which is called the streaming potential, is generated. This is the conversion of pressure into electricity. This kind of conversion provides a way to obtain electrical energy through mechanical energy during fluid flow. The energy conversion mechanism is widely used in the study of microchannel. Chakraborty et al. [28] studied the influence of hydrophobic effects on streaming potential mediated flow. Bandopadhyay and Mandalb et al. [29] studied the flow of two immiscible fluids under pressure drive. They analyzed the influence of changing the net conductivity on the concomitant streaming potential. Zhao and Jian et al. [30] studied the heat transfer characteristics under the influence of applied pressure gradient and magnetic field in a parallel-plate microchannel. Chen and Jian [31] discussed the streaming potential through microparallel channels under low zeta potential approximation conditions. They summarized the effects of the dimensionless electrokinetic width and the rotational angular velocity on the streaming potential. Ding and Jian [32] studied the flow of viscoelastic fluid under an oscillating pressure gradient and concluded the resonances that are generated for the streaming potential field and for the flow rate. In many studies, the magnetic field is often applied based on the pressure gradient. Magnetic field is widely used in microscale flow research because it has many distinct advantages, such as low manufacturing cost, low heat generation, and high flow rate [33,34,35,36].

Based on the above analysis of the advancements that have been achieved in fluid mechanics, this paper studies the streaming potential and velocity field through a microchannel under the condition that the potentials on the walls are modulated, and the pressure gradient and magnetic field are applied. Firstly, the Poisson-Boltzmann equation is established, and the analytical solution of electric potential is obtained via the superposition principle. Secondly, the modified Navier-Stokes (N-S) equation is determined according to the model conditions, and the analytical solutions for the velocity are obtained. The unknown streaming potential is involved during velocity expression. The unknown streaming potential can be calculated under the condition that the net ionic current in the solution is zero. Finally, the influences of the related parameters on the flow field, streaming potential, velocity field and flow rate are discussed in the form of graphs.

## 2. Mathematical Model

**Figure 1:**
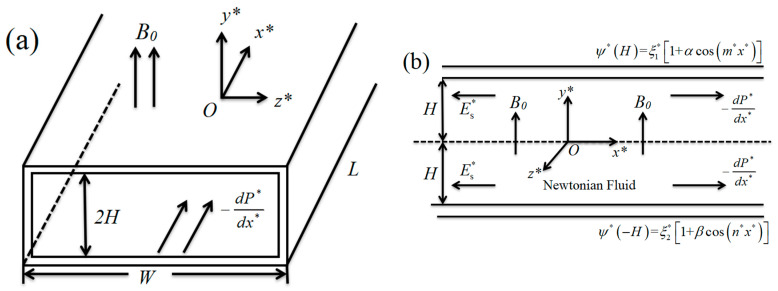
Schematic of the physical model. (**a**) 3D view of microchannel; (**b**) The cross section of the microchannel.

In this study, the streaming potential, velocity and flow rate of the Newtonian fluid under the influence of a magnetic field and a pressure gradient are considered in a parallel-plate microchannel. The schematic diagram of the physical model is depicted in Figure 1. The Newtonian fluid is assumed to be incompressible, and the walls of the microchannel are asymmetrically charge-modulated. We assume that Newtonian fluid passes through the parallel-plate microchannel with length *L*, width *W*, and height *H*, where *L* >> 2*H*, *L* >> *W*, and where the aspect ratio of *W*/*H* is very large. The Cartesian coordinate system (x*, y*, z*) is established in the center of the microchannel with *O* as the origin. The magnetic field ***B*** acts on the fluid along the y* axis, and the pressure gradient −dP*/dx* acts on the fluid along the x* axis. During the fluid flow process, there is a chemical interaction with the walls that generate the EDL. The excess ions that are generated by the flow in the electrolyte solution will gather at the downstream of the microchannel. Therefore, the streaming potential ***E*** is obtained under the drive of the magnetic field and pressure gradient, where the direction is in the negative direction of the x* axis. It can be shown that the magnetic field and the pressure gradient are the basic driving mechanisms of the subsequent fluid flow. It was assumed that the flow in the microchannel was stable throughout the entire flow process. The potential based on modulation is asymmetric, and the zeta potential of the upper and lower parallel plates can be expressed as

(1a) $$\psi^{*}{|}_{y=H}=\xi_{1}^{*}\left[1+\alpha \cos\left(m^{*}x^{*}\right)\right]$$

(1b) $$\psi^{*}{|}_{y=-H}=\xi_{2}^{*}\left[1+\beta \cos\left(n^{*}x^{*}\right)\right]$$

where ψ* is the potential distribution of the walls, *ξ*_1_* and *ξ*_2_* represent the amplitudes of the top and bottom surfaces respectively, *α* and *β* are constants and m* and n* are the patterning frequencies. Because the cosine term produces a vertical velocity, the fluid flow is considered to be two-dimensional. Because there are *L*, *W* >> 2*H* in the rectangular microchannel, it can be considered that the velocity component in the z* direction is zero.

### 2.1. EDL Potential Distribution 

We assume that the parallel-plate microchannel is filled with symmetrical electrolyte solutions. According to the formation theory of EDL, the potential ψ* distribution can be described by the following Poisson–Boltzmann (PB) equation:

(2) $$\frac{\partial^{2}\psi^{*}}{\partial x^{{*}^{2}}}+\frac{\partial^{2}\psi^{*}}{\partial y^{{*}^{2}}}=-\frac{\rho_{e}}{\varepsilon }$$

(3) $$\rho_{e}=-2n_{0}ze\sinh\left(ze\psi^{*}/k_{B}T_{a}\right)$$

where *ρ_e_* is the local volumetric net charge density, *ε* is the permittivity of the electrolyte solution, *n*_0_ is the bulk ionic concentration, *z* is the ion valence, *e* is the charge of the electron, *k_B_* is the Boltzmann constant, *T_a_* is the absolute temperature.

We suppose the electric potential is much smaller than the thermal potential, the term zeψ*/*k_B_T_a_* is less than unity. The hyperbolic sine function can be approximated by Debye-Hückel as follows [37]:

(4) $$\sinh\left(ze\psi^{*}/k_{B}T_{a}\right)\approx ze\psi^{*}/k_{B}T_{a}\;\;\mathrm{for}\;\;ze\psi^{*}/k_{B}T_{a}\ll 1$$

The definition of 1/κ* = (*εk_B_T_a_*/2*e*^2^*z*^2^*n*_0_)^1/2^ is given by using the above approximations. Additionally, the Poisson–Boltzmann equation is linearized and becomes

(5) $$\frac{\partial^{2}\psi^{*}}{\partial x^{{*}^{2}}}+\frac{\partial^{2}\psi^{*}}{\partial y^{{*}^{2}}}=\kappa^{{*}^{2}}\psi^{*}$$

where the Debye length 1/κ* is a measure of the EDL thickness and is a property of the electrolyte solution. The boundary conditions are Equation (1a,b).

Introduce the following dimensional variables:

(6) $$\begin{array}{l} \psi \;=\;\frac{\psi^{*}}{\psi_{s}},\;\left(\xi_{1},\;\xi_{2}\right)\;=\;\frac{\left(\xi_{1}^{*},\;\xi_{2}^{*}\right)}{\psi_{s}},\;\left(x,y\right)\;=\;\frac{\left(x^{*},y^{*}\right)}{H}\\ \left(m,n\right)\;=\;\left(m^{*},n^{*}\right)H,\;\kappa =\kappa^{*}H,\;\psi_{s}=\frac{k_{B}T_{a}}{ze} \end{array}$$

Through dimensionless transformation, Equation (5) is

(7) $$\frac{\partial^{2}\psi }{\partial x^{2}}+\frac{\partial^{2}\psi }{\partial y^{2}}=\kappa^{2}\psi$$

The dimensionless form of the boundary condition is

(8a) $$\psi {|}_{y=1}=\xi_{1}\left[1+\alpha \cos\left(mx\right)\right]$$

(8b) $$\psi {|}_{y=-1}=\xi_{2}\left[1+\beta \cos\left(nx\right)\right]$$

According to the superposition principle, the equation and boundary conditions are divided into three parts, so the solution of the equation can be expressed as follows [38]:

(9) $$\psi =f_{1}\left(y\right)+f_{2}\left(y\right)\cos\left(mx\right)+f_{3}\left(y\right)\cos\left(nx\right)$$

where *f*_1_, *f*_2_ and *f*_3_ can be obtained according to the corresponding boundary conditions after splitting:

(10a) $$f_{1}\left(y\right)=\frac{\xi_{1}\sinh\left[\kappa \left(1+y\right)\right]+\xi_{2}\sinh\left[\kappa \left(1-y\right)\right]}{\sinh\left(2\kappa \right)}$$

(10b) $$f_{2}\left(y\right)=\frac{\xi_{1}\alpha \sinh\left[M\left(1+y\right)\right]}{\sinh\left(2M\right)}$$

(10c) $$f_{3}\left(y\right)=\frac{\xi_{2}\beta \sinh\left[N\left(1-y\right)\right]}{\sinh\left(2N\right)}$$

(10d) $$M^{2}=m^{2}+\kappa^{2}$$

(10e) $$N^{2}=n^{2}+\kappa^{2}$$

### 2.2. Velocity Distribution

Considering the two-dimensional flow of incompressible fluid in a parallel-plate microchannel when it is under the influence of magnetic field and pressure field, the momentum equation and continuity equation of low Reynolds number limit can be expressed as:

(11) ∇·u=0

(12) $$\rho \frac{Du}{Dt^{*}}=-\nabla P^{*}+\mu \nabla^{2}u+F$$

Here, ***u*** is the velocity field, and we only need to consider the velocities in the two directions x* and y*. *ρ* is the fluid density, P* is the pressure, *μ* is the dynamic viscosity of the fluid. In addition to the pressure gradient, the net body force ***F*** also has other external forces caused by the interaction between the external magnetic field and the induced electric field:

(13) $$F=\rho_{e}E+J\times B$$

where

(14) J=σ(E+u×B)

(15) $$B=\left(0,B_{0},0\right),\;u=\left(u^{*},v^{*},0\right),\;E=\left(E_{s}^{*},0,0\right)$$

Here, ***J*** is the local ion current density satisfying Ohm’s law. *σ* is the electrical conductivity of the medium, ***E*** is the induced electric field and ***B*** is the applied magnetic field. Because the magnetic Reynolds number is small, the magnetic field is independent of the velocity. The N-S equation can be simplified to its two-dimensional component form:

(16) $$\frac{\partial \tau_{xx}^{*}}{\partial x^{*}}+\frac{\partial \tau_{yx}^{*}}{\partial y^{*}}+\rho_{e}E_{s}^{*}-\sigma u^{*}B_{0}^{2}=0$$

(17) $$\frac{\partial \tau_{xy}^{*}}{\partial x^{*}}+\frac{\partial \tau_{yy}^{*}}{\partial y^{*}}=0$$

The constitutive equation of Newtonian fluid satisfies:

(18) $$\tau_{ij}^{*}=\left[\begin{array}{cc} \tau_{xx}^{*} & \tau_{xy}^{*}\\ \tau_{yx}^{*} & \tau_{yy}^{*} \end{array}\right]=\left[\begin{array}{cc} -P^{*}+2\mu \frac{\partial u^{*}}{\partial x^{*}} & \mu \left(\frac{\partial u^{*}}{\partial y^{*}}+\frac{\partial v^{*}}{\partial x^{*}}\right)\\ \mu \left(\frac{\partial u^{*}}{\partial y^{*}}+\frac{\partial v^{*}}{\partial x^{*}}\right) & -P^{*}+2\mu \frac{\partial v^{*}}{\partial y^{*}} \end{array}\right]$$

where τ_ij_* is the stress tensor, in which τ_ij_* is eliminated in combination with Equations (11), (16)–(18) and then simplified to obtain the final governing equation:

(19) $$\frac{\partial u^{*}}{\partial x^{*}}+\frac{\partial v^{*}}{\partial y^{*}}=0$$

(20) $$-\frac{\partial P^{*}}{\partial x^{*}}+\mu \left(\frac{\partial^{2}u^{*}}{\partial x^{{*}^{2}}}+\frac{\partial^{2}u^{*}}{\partial y^{{*}^{2}}}\right)+\rho_{e}E_{s}^{*}-\sigma u^{*}B_{0}^{2}=0$$

(21) $$-\frac{\partial P^{*}}{\partial y^{*}}+\mu \left(\frac{\partial^{2}v^{*}}{\partial y^{{*}^{2}}}+\frac{\partial^{2}v^{*}}{\partial x^{{*}^{2}}}\right)=0$$

The boundary conditions that satisfy the influence of slip and no penetration are as follows [39]:

(22) $$u^{*}+\delta^{*}\frac{\partial u^{*}}{\partial y^{*}}{|}_{y^{*}=H}=0,\;u^{*}-\delta^{*}\frac{\partial u^{*}}{\partial y^{*}}{|}_{y^{*}=-H}=0,\;v^{*}{|}_{y^{*}=\pm H}=0$$

where δ* is the slip length, the following dimensional variables are introduced:

(23) $$\begin{array}{l} \left(x,y\right)=\frac{\left(x^{*},y^{*}\right)}{H},\;Ha=B_{0}H\sqrt{\frac{\sigma }{\mu }},\;\delta =\frac{\delta^{*}}{H}\\ P=\frac{P^{*}}{P_{0}},\;\left(u,v\right)=\frac{\left(u^{*},v^{*}\right)}{u_{p}},\;u_{e}=\frac{\varepsilon E_{0}\psi_{s}}{\mu }\\ E_{s}=\frac{E_{s}^{*}}{E_{0}},\;u_{r}=\frac{u_{e}}{u_{p}},\;u_{p}=\frac{HP_{0}}{\mu },\;\kappa =\kappa^{*}H \end{array}$$

where *u_p_* is the characteristic velocity of the fluid flow driven by pressure, *u_r_* is the characteristic velocity of the electric flow, *δ* is the nondimensional slip length, *P*_0_ is the characteristic pressure, *Ha* is the Hartmann number, *E*_0_ is the characteristic scale of the electric field. After the dimensionless transformation, Equations (19)–(21) and boundary condition Equation (22) become

(24) $$\frac{\partial u}{\partial x}+\frac{\partial v}{\partial y}=0$$

(25) $$-\frac{\partial P}{\partial x}+\frac{\partial^{2}u}{\partial x^{2}}+\frac{\partial^{2}u}{\partial y^{2}}-\kappa^{2}E_{s}u_{r}\psi -Ha^{2}u=0$$

(26) $$-\frac{\partial P}{\partial y}+\frac{\partial^{2}v}{\partial x^{2}}+\frac{\partial^{2}v}{\partial y^{2}}=0$$

(27) $$u+\delta \frac{\partial u}{\partial y}{|}_{y=1}=0,\;u-\delta \frac{\partial u}{\partial y}{|}_{y=-1}=0,\;v{|}_{y=\pm 1}=0$$

The stream function is defined based on Equation (24) [40]:

(28) $$u=\frac{\partial \varphi }{\partial y},\;v=-\frac{\partial \varphi }{\partial x}$$

Combining Equations (25) and (26) to eliminate the pressure *P*, an equation that is related to the stream function is obtained:

(29) $$2\frac{\partial^{4}\varphi }{\partial y^{2}\partial x^{2}}+\frac{\partial^{4}\varphi }{\partial y^{4}}+\frac{\partial^{4}\varphi }{\partial x^{4}}-Ha^{2}\frac{\partial^{2}\varphi }{\partial y^{2}}-\kappa^{2}E_{s}u_{r}\frac{\partial \psi }{\partial y}=0$$

The boundary conditions become

(30) $$\begin{array}{ll} \frac{\partial \varphi }{\partial y}+\delta \frac{\partial^{2}\varphi }{\partial y^{2}}{|}_{y=1}=0, & \frac{\partial \varphi }{\partial y}-\delta \frac{\partial^{2}\varphi }{\partial y^{2}}{|}_{y=-1}=0\\ \frac{\partial \varphi }{\partial x}{|}_{y=1}=0, & \frac{\partial \varphi }{\partial x}{|}_{y=-1}=0 \end{array}$$

Additionally, using the superposition principle, the solution of Equation (29) satisfies the following form:

(31) $$\varphi =g_{1}\left(y\right)+g_{2}\left(y\right)\cos\left(mx\right)+g_{3}\left(y\right)\cos\left(nx\right)$$

Through complex calculations and combined with boundary conditions, three polynomials *g*_1_, *g*_2_ and *g*_3_ which are related to *y* can be determined as follows:

(32a) $$g_{1}\left(y\right)=A_{11}\cosh\left[\kappa \left(1+y\right)\right]+A_{12}\cosh\left[\kappa \left(1-y\right)\right]+\gamma_{1}\exp(Hay)+\gamma_{2}\exp(-Hay)+\gamma_{3}+\gamma_{4}y$$

(32b) $$g_{2}\left(y\right)=A_{2}\cosh\left[M\left(1+y\right)\right]+\gamma_{5}\exp(\lambda_{21}y)+\gamma_{6}\exp(\lambda_{22}y)+\gamma_{7}\exp(\lambda_{23}y)+\gamma_{8}\exp(\lambda_{24}y)$$

(32c) $$g_{3}\left(y\right)=A_{3}\cosh\left[N\left(1-y\right)\right]+\gamma_{9}\exp(\lambda_{31}y)+\gamma_{10}\exp(\lambda_{32}y)+\gamma_{11}\exp(\lambda_{33}y)+\gamma_{12}\exp(\lambda_{34}y)$$

The coefficients in Equation (32a,b,c) are expressed as follows:

(33a) $$A_{11}=\frac{\left[\kappa^{3}E_{s}u_{r}\left(1-\kappa^{2}+Ha^{2}\right)\right]\xi_{1}}{Ha^{2}\sinh\left(2\kappa \right)\left(\kappa^{2}-Ha^{2}\right)}$$

(33b) $$A_{12}=-\frac{\left[\kappa^{3}E_{s}u_{r}\left(1-\kappa^{2}+Ha^{2}\right)\right]\xi_{2}}{Ha^{2}\sinh\left(2\kappa \right)\left(\kappa^{2}-Ha^{2}\right)}$$

(33c) $$A_{2}=\frac{\kappa^{2}E_{s}u_{r}\xi_{1}\alpha M}{\sinh\left(2M\right)\left(M^{2}-\lambda_{22}^{2}\right)\left(M^{2}-\lambda_{24}^{2}\right)}$$

(33d) $$A_{3}=-\frac{\kappa^{2}E_{s}u_{r}\xi_{2}\beta N}{\sinh\left(2N\right)\left(N^{2}-\lambda_{32}^{2}\right)\left(N^{2}-\lambda_{34}^{2}\right)}$$

where *λ_ij_* (*i* = 2, 3; *j* = 1, 2, 3, 4) satisfies the following two equations, and we can conclude that *λ*_21_ = −*λ*_22_, *λ*_23_ = −*λ*_24_, *λ*_31_ = −*λ*_32_, *λ*_33_ = −*λ*_34_.

(34a) $$\lambda_{2j}^{4}-\left(2m^{2}+Ha^{2}\right)\lambda_{2j}^{2}+m^{4}=0$$

(34b) $$\lambda_{3j}^{4}-\left(2n^{2}+Ha^{2}\right)\lambda_{3j}^{2}+n^{4}=0$$

Because the smallest part of this Equation (29) is the partial derivative of the second order, we can assume that *γ*_3_ = 0 and *γ*_4_ = 1. In addition, the rest of *γ_k_* (*k* = 1, 2, 5–12) can represented by the following matrix equation:

(35) $${\hat{\Gamma }}_{l}{\hat{\gamma }}_{l}={\hat{X}}_{l}\;\;l=1,2,3$$

The specific matrix of the equation is shown in Appendix A. Using these coefficients, we can determine the stream function that is needed before we can solve for the velocity. 

### 2.3. Streaming Potential

Through the previous calculations, we are able to obtain the analytical solution for the velocity when it is under the joint action of pressure and magnetic field. However, the expression still contains the unknown streaming potential *E_s_* which needs to be determined. Because there is no applied electric field, the streaming potential can be determined by considering the condition that the net ion current in the electrolyte solution is zero. When the fluid reaches a stable state, it satisfies the following equation.

(36) $$I=\int_{-H}^{H}ze\left(u^{+}n^{+}-u^{-}n^{-}\right)dy^{*}=0$$

where *u*^±^ is the velocity of cation and anion in the x* direction, and *n*^±^ is the concentration of cation and anion, which satisfy the following relationship respectively.

(37) $$u^{\pm }=u^{*}\pm \frac{ezE_{s}^{*}}{f}$$

(38) $$n^{\pm }=n_{0}\exp\left(\mp ez\psi^{*}/k_{B}T_{a}\right)$$

where ƒ is the friction coefficient of the ions. Additionally, a new parameter, *R* = 2*e*^2^*z*^2^*µ*/*εk_B_T_a_f*, is introduced, which is a dimensionless parameter that is equivalent to the ionic Peclet number [41]. Substituting the above parameters into Equation (36), the following equation can be obtained:

(39) $$\int_{-1}^{1}u\psi dy=RE_{s}u_{r}$$

After calculation, the expression of the dimensionless streaming potential is

(40) $$E_{s}=\frac{T_{1}}{u_{r}\left(R-T_{2}\right)}$$

The coefficients that are involved in the equation are shown in Appendix B. On this basis, the flow rate per unit width in the *z* direction is considered through the cross section at *x* = 0, and the flow rate *Q* can be calculated by $\int_{-1}^{1}udy$.

## 3. Result and Discussion

The analytical solutions of velocity and streaming potential are obtained by calculating the pressure gradient and the magnetic field in the parallel-plate microchannel. Next, we consider the ranges of values of the relevant parameters in order to determine the required dimensionless parameters. Let the half height of the microchannel *H* is about 200 μm, the density of the fluid *ρ* is about 10^−3^ kg/m^3^, the dynamic viscosity *μ* is about 10^−3^ kg/(m·s), the range of conductivity *σ* is 2.2 × 10^−4^~10^2^ S/m [42], the strength of the external magnetic field *B*_0_ is 0.01~5 T. According to *Ha* = *B*_0_*H*(*σ*/*μ*)^1/2^, the range of the Hartmann number (*Ha*) can be obtained from 0 to 0.4 with *Ha* = 1 as the maximum permissible upper limit [43,44] theoretically. Based on the previous theoretical derivation, in order to satisfy the Debye–Hückel linearization approximation conditions, the dimensionless zeta potential should satisfy *ψ* ≤ 1. In the following discussion about the upper and lower surface mode potentials, the ranges of the amplitudes (*ξ*_1_ and *ξ*_2_), constants (*α* and *β*) and mode frequencies (*m* and *n*) are 0~0.2, 0~6 and 0~6 respectively. When *α* = *β* = 0, the zeta potential are constants. The values of *R* and *u_r_* are assumed to be *R*~0.3–1 and *u_r_*~0.1–1 when in the dimensionless form. 

### 3.1. Flow Field

**Figure 2:**
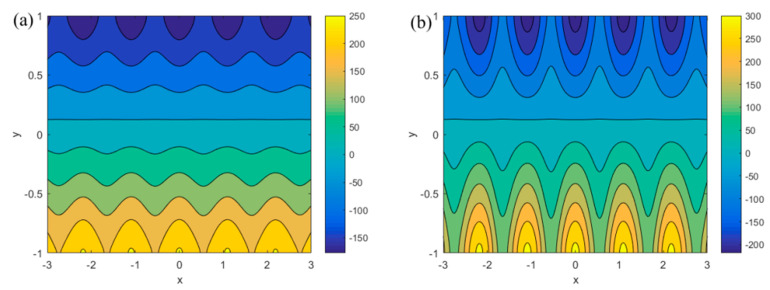
Distributions of streamlines for different *α* and *β* (*Ha* = 1, *κ* = 8, *δ* = 0, *u_r_* = 0.6, *R* = 0.6, *m* = 5.5, *n* = 5.8, *ξ*_1_ = 0.15, *ξ*_2_ = 0.18). (**a**) *α* = 1.5, *β* = 1.8; (**b**) *α* = 5.5, *β* = 5.8.

**Figure 3:**
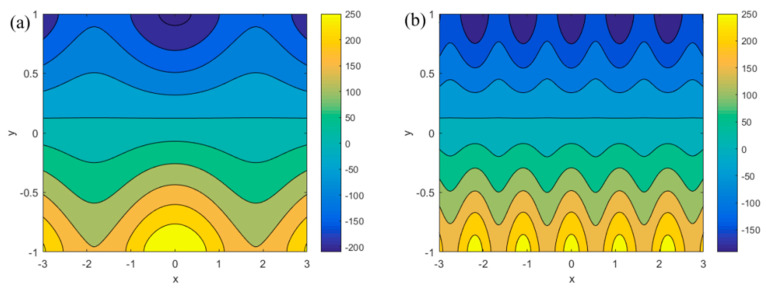
Changes in streamlines for different *m* and *n* (*Ha* = 1, *κ* = 8, *δ* = 0, *u_r_* = 0.6, *R* = 0.6, *α* = 3, *β* = 3, *ξ*_1_ = 0.15, *ξ*_2_ = 0.18). (**a**) *m* = 1.5, *n* = 1.8; (**b**) *m* = 5.5, *n* = 5.8.

According to the expression of the zeta potentials (Equation (1a,b)), the influences of the modulated charged surfaces on the flow field are described in Figure 2 and Figure 3. It can be seen that periodic cyclic flow is generated due to modulated potentials. The reverse flow induced in the microchannel changes the positive direction velocity into negative direction, resulting in an eddy current.

Figure 2 studies the influence of *α* (*α* = 1.5, 5.5) and *β* (*β* = 1.8, 5.8) on the flow field when *Ha* = 1, *κ* = 8, *δ* = 0, *u_r_* = 0.6, *R* = 0.6, *m* = 5.5, *n* = 5.8, *ξ*_1_ = 0.15, *ξ*_2_ = 0.18. Because of the asymmetry of the wall potentials, it can be seen from the Figure 2 that the streamlines near the upper and lower walls are also asymmetric. As the values of parameters *α* and *β* increase, the streamlines that can be observed in Figure 2b are denser than the ones seen in Figure 2a. Additionally, the characteristic of the vortexes that are near the walls in Figure 2b are significant. This means that constants *α* and *β* are the main elements that control the strength of the vortexes.

Figure 3 shows the flow field distribution at *Ha* = 1, *κ* = 8, *δ* = 0, *u_r_* = 0.6, *R* = 0.6, *α* = 3, *β* = 3, *ξ*_1_ = 0.15, *ξ*_2_ = 0.18 when the mode frequencies *m* (*m* = 1.5, 5.5) and *n* (*n* = 1.8, 5.8) change. It can be observed in the Figure 3a,b that with the increase in mode frequencies, the density of streamlines become less obvious. However, along the direction of the *x*-axis, it can be seen that the periodicity becomes more pronounced as *m* and *n* increase. The reason for this phenomenon is that the cos(*mx*) and cos(*nx*) in Equation (1a,b) of the zeta potentials play important roles. The existence of cosine terms produces vertical velocity in the *y*-axis direction, leading to the appearance of vortexes. When *m* and *n* are larger, the 2*π*/*m* and 2*π*/*n* periods are smaller. This means that *m* and *n* are the main elements that control the periodicity of the eddy currents.

### 3.2. Analysis of the Streaming Potential

**Figure 4:**
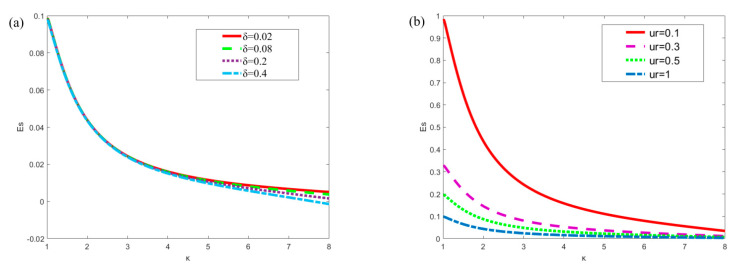
Variation of the dimensionless streaming potential *E_s_* with the dimensionless parameter *κ* for different values of *δ* and *u_r_* (*x* = *π*/4, *m* = 0.5, *n* = 0.8, *ξ*_1_ = 0.02, *ξ*_2_ = 0.02, *α* = 5, *β* = 5, *R* = 1, *Ha* = 1). (**a**) *E_s_* at different *δ* (*u_r_* = 1); (**b**) *E_s_* at different *u_r_* (*δ* = 0.1).

Figure 4a,b respectively show the influence of slip length *δ* (*δ* = 0.02, 0.08, 0.2, 0.4) and *u_r_* (*u_r_* = 0.1, 0.3, 0.5, 1) on the streaming potential when *x* = *π*/4, *m* = 0.5, *n* = 0.8, *ξ*_1_ = 0.02, *ξ*_2_ = 0.02, *α* = 5, *β* = 5, *R* = 1, *Ha* = 1. In Figure 4a, when *κ* is small, the slip length does not affect the streaming potential. When *κ* > 4, the streaming potential decreases slightly with the increase of slip length. In Figure 4b, the influence of *u_r_* is obvious, and the streaming potential decreases with the increase of *u_r_*. The reason for this phenomenon can be explained from the perspective of physical significance. According to the equation *u_r_* = *u_e_*/*u_p_*, when *u_r_* increases, *u_p_* will decrease. A diminution in *u_p_* means that the influence of the pressure gradient is weakened, resulting in a corresponding abatement in the streaming potential. This conclusion can also be drawn from Equation (40). On the other hand, with the increase of *κ*, the influence of *u_r_* decreases gradually.

**Figure 5:**
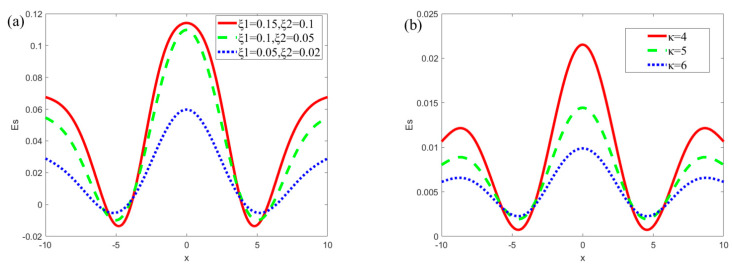
The variations of streaming potential *E_s_* for different *ξ_i_* (*i* = 1, 2) and *κ* (*m* = 0.5, *n* = 0.8, *α* = 5, *β* = 5, *R* = 1, *δ* = 0.1, *Ha* = 1, *u_r_* = 1). (**a**) *E_s_* at different *ξ*_1_ and *ξ*_2_ (*κ* = 3); (**b**) *E_s_* at different *κ* (*ξ*_1_ = 0.02, *ξ*_2_ = 0.02).

Figure 5 describes the variations in the streaming potential *E_s_* for different *ξ_i_* (*i* = 1, 2) and *κ* when *m* = 0.5, *n* = 0.8, *α* = 5, *β* = 5, *R* = 1, *δ* = 0.1, *Ha* = 1, *u_r_* = 1. In Figure 5a, the relationship between the zeta potential and the streaming potential can be analyzed by changing the amplitude of the mode potential. As the amplitude of the mode potential become more enhanced, the wall zeta potential increases, resulting in an increase in the potential in the electrolyte solution. It can be seen from Figure 5a that the streaming potential increases as the amplitude heightens. In terms of the generation mechanism of streaming potential, the increase in potential leads to an increase in the proportion of positive and negative ions that is present in the solution, while a difference in the number of positive and negative ions in the electrolyte solution is positively correlated with the streaming potential. As such, the streaming potential is positively correlated with the potential on the walls. The plot oscillation along the *x*-axis is caused by the emergence of the vertical velocity due to the modulated surface potential. In Figure 5b, the streaming potential decreases with the increase of the *κ* (*κ* = 4, 5, 6). As *κ* increases, the thickness of the EDL decreases, leading to a decrease in the number of ions in the EDL, and thus the induced streaming potential generated by pressure gradient decreases gradually.

### 3.3. Analysis of Dimensionless Velocity

**Figure 6:**
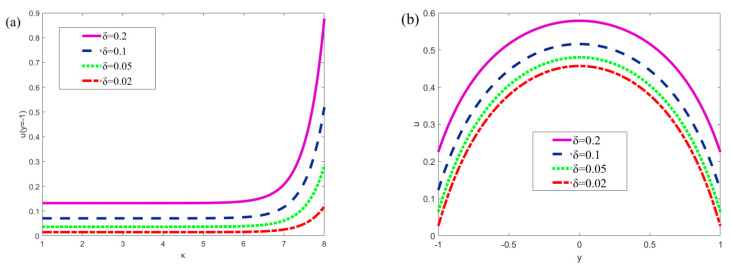
The variations of velocity with the nondimensional slip length *δ* (*x* = *π*/2, *m* = 0.5, *n* = 0.8, *Ha* = 1, *ξ*_1_ = 0.02, *ξ*_2_ = 0.02, *α* = 5, *β* = 5, *u_r_* = 1, *R* = 1). (**a**) The variations in velocity at *y* = −1; (**b**) Velocity variations in microchannel (*κ* = 5).

Figure 6 describes the influence of the slip length on the velocity *u* at the wall surface (*y* = −1) and in the entire microchannel. It can be seen from Figure 6a that an increase in slip length will lead to an increase in the wall velocity *u*. Figure 6b demonstrates that the same phenomenon exists in the whole microchannel. The reason for this phenomenon is that the existence of slip length is equivalent to the application of a nonzero initial velocity to the fluid, which will also affect the fluid in the whole microchannel.

**Figure 7:**
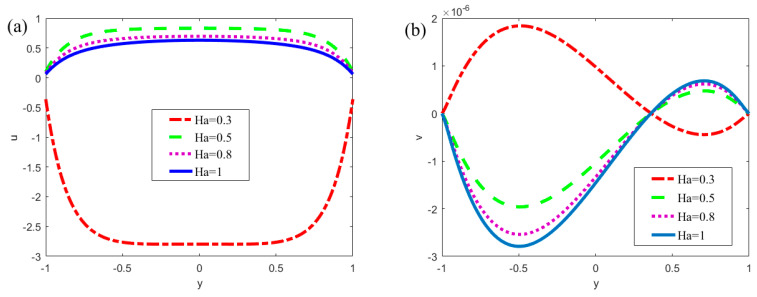
The dimensionless velocity at different positions varies with the Hartmann number *Ha* (*x* = *π*/4, *m* = 0.5, *n* = 0.8, *κ* = 7, *α* = 5, *β* = 5, *ξ*_1_ = 0.02, *ξ*_2_ = 0.02, *δ* = 0.02, *u_r_* = 1, *R* = 1). (**a**) velocity *u*; (**b**) velocity *v*.

Figure 7 describes the influence of the Hartmann number (*Ha* = 0.3, 0.5, 0.8, 1) on the velocities, which are based on *x* = *π*/4, *m* = 0.5, *n* = 0.8, *κ* = 7, *α* = 5, *β* = 5, *ξ*_1_ = 0.02, *ξ*_2_ = 0.02, *δ* = 0.02, *u_r_* = 1, *R* = 1. It can be seen from Figure 7a that the velocity *u* decreases with the increase of *Ha*. Additionally, the velocity changes rapidly near the walls. Under the influence of the modulated potentials, the vortexes and oscillations generate in the velocity profile. The negative values maybe emerge in the velocity profile (when *Ha* = 0.3 in Figure 7a), which mean the backflow of the fluid. According to the definition of the Hartman number, which is the ratio between the electromagnetic force and the viscous force in physics, and only one term in the modified N-S equation contains magnetic field, which corresponds to −*Ha*^2^*u* in the dimensionless Equation (25), it can be seen that the Hartman number plays an obstructive role in the fluid movement process. An opposite trend can be observed in Figure 7b. In Figure 7b, the velocity *v* increases with the increase of *Ha*. The reason for this phenomenon is that the flow rate in the parallel plate is a certain amount, when the Hartmann number increases, the velocity component *u* of the *x*-direction decreases, resulting in the velocity component *v* of the *y*-direction increasing.

**Figure 8:**
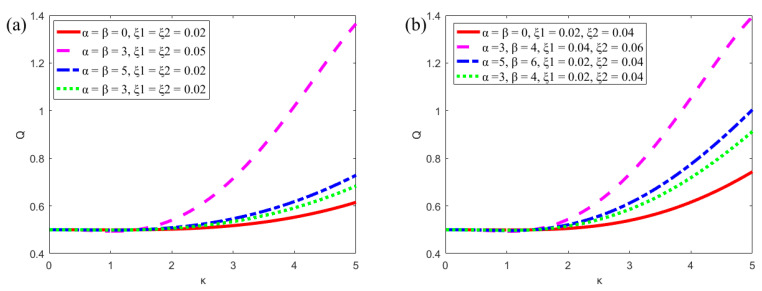

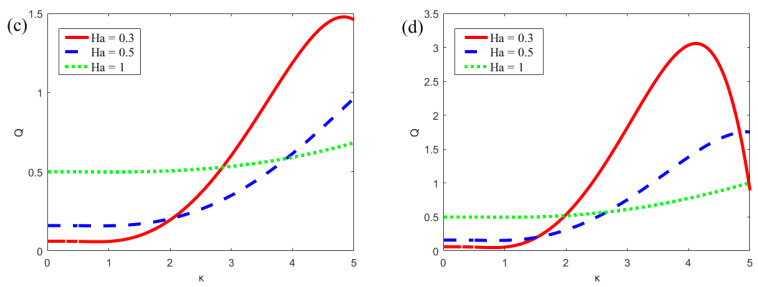
The variations of the flow rate in microchannel of the parallel plate (*δ* = 0.02, *u_r_* = 1, *R* = 1). (**a**) Symmetric modulated potentials with different *α*, *β*, *ξ*_1_ and *ξ*_2_ (*m* = *n* = 0.5, *Ha* = 1); (**b**) Asymmetric modulated potentials with different *α*, *β*, *ξ*_1_ and *ξ*_2_ (*m* = 0.5, *n* = 0.8, *Ha* = 1). (**c**) Symmetric modulated potentials with different *Ha* (*m* = *n* = 0.5, *ξ*_1_ = *ξ*_2_ = 0.02, *α* = *β* = 5); (**d**) Asymmetric modulated potentials with different *Ha* (*m* = 0.5, *n* = 0.8, *ξ*_1_ = 0.02, *ξ*_2_ = 0.04, *α* = 5, *β* = 6).

Figure 8 shows the plots of the flow rate in the microchannel with different parameters (*α*, *β*, *ξ*_1_, *ξ*_2_, *Ha*). Symmetrical (in Figure 8a,c) and asymmetric modulated (in Figure 8b,d) potentials are set at the upper and lower plates, respectively. In Figure 8a,b, when *α* = *β* = 0, the potential on surfaces are uniform. By increasing the amplitude (*ξ*_1_ and *ξ*_2_) and constant (*α* and *β*) of the modulated potentials, the corresponding flow rate also tends to increase. In Figure 8c,d, when Hartmann number is small (*Ha* = 0.3), the plot of the flow rate varies rapidly with the parameter κ. It can be found that the flow rate under the modulated potentials is larger than that under the uniform potentials.

## 4. Conclusions

In this study, the streaming potential, velocity field and flow rate of the fluid in the parallel-plate microchannel are considered under the longitudinal pressure gradient and the vertical magnetic field. Because modulated surface potentials exist on both the upper and lower walls, vertical velocity will be generated during the flow process. Therefore, the flow is two-dimensional. In this case, vortexes will appear in the microchannel, and the streamlines, streaming potential and velocity field all have the characteristics of oscillation and periodicity. The intensity and period of the eddy current will become more obvious when the modulated potentials of the walls increase (changing the value of *m*, *n*, *ξ*_1_, *ξ*_2_, *α* and *β*). Additionally, the streaming potential and flow rate increase as the modulated potentials increase. In the analysis of the relationship between the modulated potentials and the flow rate, three types of uniform potentials, symmetric modulated potentials and asymmetric modulated potentials are considered. When comparing these three cases, it can be found that the flow rate in the charge-modulated mode is larger than that in the uniform mode. This proves that modulated charged surfaces are beneficial for fluid transport and mixing. The influence of some non-dimensional parameters (Hartmann number *Ha*, slip length *δ*, dimensionless parameter *u_r_* and *κ*) are also discussed under the charge-modulated potentials. The main function of slip length *δ* is to add an initial velocity to the fluid at the walls, so the velocity of fluid increases with the increasing of slip length. Although the velocity is oscillating, the Hartman number *Ha* always hinders the flow of fluid.

## Appendix A

(A1)
$${\hat{\Gamma }}_{1}=\left(\begin{array}{cc} \left(Ha+\delta Ha^{2}\right)\exp\left(Ha\right) & -\left(Ha-\delta Ha^{2}\right)\exp\left(-Ha\right)\\ \left(Ha-\delta Ha^{2}\right)\exp\left(-Ha\right) & -\left(Ha+\delta Ha^{2}\right)\exp\left(Ha\right) \end{array}\right),$$

(A2)
$${\hat{\Gamma }}_{2}=\left[\begin{array}{cccc} \exp\left(\lambda_{21}\right) & \exp\left(\lambda_{22}\right) & \exp\left(\lambda_{23}\right) & \exp\left(\lambda_{24}\right)\\ \exp\left(-\lambda_{21}\right) & \exp\left(-\lambda_{22}\right) & \exp\left(-\lambda_{23}\right) & \exp\left(-\lambda_{24}\right)\\ \left(\lambda_{21}+\delta \lambda_{21}^{2}\right)\exp\left(\lambda_{21}\right) & \left(\lambda_{22}+\delta \lambda_{22}^{2}\right)exp\left(\lambda_{22}\right) & \left(\lambda_{23}+\delta \lambda_{23}^{2}\right)\exp\left(\lambda_{23}\right) & \left(\lambda_{24}+\delta \lambda_{24}^{2}\right)\exp\left(\lambda_{24}\right)\\ \left(\lambda_{21}-\delta \lambda_{21}^{2}\right)\exp\left(-\lambda_{21}\right) & \left(\lambda_{22}-\delta \lambda_{22}^{2}\right)\exp\left(-\lambda_{22}\right) & \left(\lambda_{23}-\delta \lambda_{23}^{2}\right)\exp\left(-\lambda_{23}\right) & \left(\lambda_{24}-\delta \lambda_{24}^{2}\right)\exp\left(-\lambda_{24}\right) \end{array}\right]$$

(A3)
$${\hat{\Gamma }}_{3}=\left[\begin{array}{cccc} \exp\left(\lambda_{31}\right) & \exp\left(\lambda_{32}\right) & \exp\left(\lambda_{33}\right) & \exp\left(\lambda_{34}\right)\\ \exp\left(-\lambda_{31}\right) & \exp\left(-\lambda_{32}\right) & \exp\left(-\lambda_{33}\right) & \exp\left(-\lambda_{34}\right)\\ \left(\lambda_{31}+\delta \lambda_{31}^{2}\right)\exp\left(\lambda_{31}\right) & \left(\lambda_{32}+\delta \lambda_{32}^{2}\right)\exp\left(\lambda_{32}\right) & \left(\lambda_{33}+\delta \lambda_{33}^{2}\right)\exp\left(\lambda_{33}\right) & \left(\lambda_{34}+\delta \lambda_{34}^{2}\right)\exp\left(\lambda_{34}\right)\\ \left(\lambda_{31}-\delta \lambda_{31}^{2}\right)\exp\left(-\lambda_{31}\right) & \left(\lambda_{32}-\delta \lambda_{32}^{2}\right)\exp\left(-\lambda_{32}\right) & \left(\lambda_{33}-\delta \lambda_{33}^{2}\right)\exp\left(-\lambda_{33}\right) & \left(\lambda_{34}-\delta \lambda_{34}^{2}\right)\exp\left(-\lambda_{34}\right) \end{array}\right]$$

(A4)
$${\hat{\gamma }}_{1}=\left(\begin{array}{c} \gamma_{1}\\ \gamma_{2} \end{array}\right),{\hat{\gamma }}_{2}=\left(\begin{array}{c} \gamma_{5}\\ \gamma_{6}\\ \gamma_{7}\\ \gamma_{8} \end{array}\right),{\hat{\gamma }}_{3}=\left(\begin{array}{c} \gamma_{9}\\ \gamma_{10}\\ \gamma_{11}\\ \gamma_{12} \end{array}\right)$$

(A5)
$${\hat{X}}_{1}=\left(\begin{array}{c} -A_{11}\kappa \sinh\left(2\kappa \right)-\delta A_{11}\kappa^{2}\cosh\left(2\kappa \right)-\delta A_{12}\kappa^{2}-1\\ A_{12}\kappa \sinh\left(2\kappa \right)+\delta A_{11}\kappa^{2}+\delta A_{12}\kappa^{2}\cosh\left(2\kappa \right)-1 \end{array}\right)$$

(A6)
$${\hat{X}}_{2}=\left(\begin{array}{c} -A_{2}\cosh\left(2M\right)\\ -A_{2}\\ -A_{2}\left[M\sinh\left(2M\right)+\delta M^{2}\cosh\left(2M\right)\right]\\ A_{2}\delta M^{2} \end{array}\right)$$

(A7)
$${\hat{X}}_{3}=\left(\begin{array}{c} -A_{3}\\ -A_{3}\cosh\left(2N\right)\\ -A_{3}\delta N^{2}\\ A_{3}\left[N\sinh\left(2N\right)+\delta N^{2}\cosh\left(2N\right)\right] \end{array}\right)$$

## Appendix B

(A8)
$$T_{1}=T_{11}+T_{12}+T_{13}$$

(A9)
$$T_{11}=\frac{2\left(\xi_{1}+\xi_{2}\right)\sinh^{2}\left(\kappa \right)}{\sinh\left(2\kappa \right)\kappa }$$

(A10)
$$T_{12}=\frac{2\xi_{1}\alpha \sinh^{2}\left(M\right)}{M\sinh\left(2M\right)}\cos\left(mx\right)$$

(A11)
$$T_{13}=\frac{2\xi_{2}\beta \sinh^{2}\left(N\right)}{N\sinh\left(2N\right)}\cos\left(nx\right)$$

(A12)
$$T_{2}=\sum_{i=1}^{9}T_{2i}$$

(A13)
$$T_{21}=\frac{\left(\xi_{1}{\bar{A}}_{11}-\xi_{2}{\bar{A}}_{12}\right)\kappa }{\sinh\left(2\kappa \right)}B_{\kappa }+\frac{\left(\xi_{2}{\bar{A}}_{11}-\xi_{1}{\bar{A}}_{12}\right)\kappa }{\sinh\left(2\kappa \right)}C_{\kappa }+\frac{HaF_{1\cdot 1\cdot 2\cdot 2}}{\sinh\left(2\kappa \right)}D_{Ha\cdot \kappa }+\frac{HaF_{2\cdot 1\cdot 1\cdot 2}}{\sinh\left(2\kappa \right)}E_{Ha\cdot \kappa }$$

(A14)
$$\begin{array}{ll} T_{22}= & [\frac{\xi_{1}{\bar{A}}_{2}M}{\sinh\left(2\kappa \right)}G_{\kappa \cdot M}+\frac{\xi_{2}{\bar{A}}_{2}M}{\sinh\left(2\kappa \right)}H_{\kappa \cdot M}+\frac{F_{2\cdot 6\cdot 1\cdot 5}\lambda_{22}}{\sinh\left(2\kappa \right)}E_{\lambda_{22}\cdot \kappa }+\frac{F_{1\cdot 6\cdot 2\cdot 5}\lambda_{22}}{\sinh\left(2\kappa \right)}D_{\lambda_{22}\cdot \kappa }+\frac{F_{2\cdot 8\cdot 1\cdot 7}\lambda_{24}}{\sinh\left(2\kappa \right)}E_{\lambda_{24}\cdot \kappa }\\ & +\frac{F_{1\cdot 8\cdot 2\cdot 7}\lambda_{24}}{\sinh\left(2\kappa \right)}D_{\lambda_{24}\cdot \kappa }]\cos\left(mx\right) \end{array}$$

(A15)
$$\begin{array}{ll} T_{23}= & [-\frac{\xi_{1}{\bar{A}}_{3}N}{\sinh\left(2\kappa \right)}H_{\kappa \cdot N}-\frac{\xi_{2}{\bar{A}}_{3}N}{\sinh\left(2\kappa \right)}G_{\kappa \cdot N}+\frac{F_{2\cdot 10\cdot 1\cdot 9}\lambda_{32}}{\sinh\left(2\kappa \right)}E_{\lambda_{32}\cdot \kappa }+\frac{F_{1\cdot 10\cdot 2\cdot 9}\lambda_{32}}{\sinh\left(2\kappa \right)}D_{\lambda_{32}\cdot \kappa }+\frac{F_{2\cdot 12\cdot 1\cdot 11}\lambda_{34}}{\sinh\left(2\kappa \right)}E_{\lambda_{34}\cdot \kappa }\\ & +\frac{F_{1\cdot 12\cdot 2\cdot 11}\lambda_{34}}{\sinh\left(2\kappa \right)}D_{\lambda_{34}\cdot \kappa }]\cos\left(nx\right) \end{array}$$

(A16)
$$T_{24}=[\frac{\xi_{1}\alpha {\bar{A}}_{11}\kappa }{\sinh\left(2M\right)}G_{\kappa \cdot M}-\frac{\xi_{1}\alpha {\bar{A}}_{12}\kappa }{\sinh\left(2M\right)}H_{\kappa \cdot M}+\frac{\xi_{1}\alpha Ha{\bar{\gamma }}_{1}}{\sinh\left(2M\right)}D_{Ha\cdot M}-\frac{\xi_{1}\alpha Ha{\bar{\gamma }}_{2}}{\sinh\left(2M\right)}E_{Ha\cdot M}]\cos\left(mx\right)$$

(A17)
$$\begin{array}{ll} T_{25}= & [\frac{\xi_{1}\alpha {\bar{A}}_{2}M}{\sinh\left(2M\right)}B_{M}+\frac{\xi_{1}\alpha {\bar{\gamma }}_{5}\lambda_{21}}{\sinh\left(2M\right)}D_{\lambda_{21}\cdot M}+\frac{\xi_{1}\alpha {\bar{\gamma }}_{6}\lambda_{22}}{\sinh\left(2M\right)}D_{\lambda_{22}\cdot M}+\frac{\xi_{1}\alpha {\bar{\gamma }}_{7}\lambda_{23}}{\sinh\left(2M\right)}D_{\lambda_{23}\cdot M}\\ & +\frac{\xi_{1}\alpha {\bar{\gamma }}_{8}\lambda_{24}}{\sinh\left(2M\right)}D_{\lambda_{24}\cdot M}]\cos^{2}\left(mx\right) \end{array}$$

(A18)
$$\begin{array}{ll} T_{26}= & [-\frac{\xi_{1}\alpha {\bar{A}}_{3}N}{\sinh\left(2M\right)}H_{M\cdot N}+\frac{\xi_{1}\alpha {\bar{\gamma }}_{9}\lambda_{31}}{\sinh\left(2M\right)}D_{\lambda_{31}\cdot M}+\frac{\xi_{1}\alpha {\bar{\gamma }}_{10}\lambda_{32}}{\sinh\left(2M\right)}D_{\lambda_{32}\cdot M}+\frac{\xi_{1}\alpha {\bar{\gamma }}_{11}\lambda_{33}}{\sinh\left(2M\right)}D_{\lambda_{33}\cdot M}\\ & +\frac{\xi_{1}\alpha {\bar{\gamma }}_{12}\lambda_{34}}{\sinh\left(2M\right)}D_{\lambda_{34}\cdot M}]\cos\left(mx\right)\cos\left(nx\right) \end{array}$$

(A19)
$$T_{27}=[\frac{\xi_{2}\beta {\bar{A}}_{11}\kappa }{\sinh\left(2N\right)}H_{\kappa \cdot N}-\frac{\xi_{2}\beta {\bar{A}}_{12}\kappa }{\sinh\left(2N\right)}G_{\kappa \cdot N}+\frac{\xi_{2}\beta Ha{\bar{\gamma }}_{1}}{\sinh\left(2N\right)}E_{Ha\cdot N}-\frac{\xi_{2}\beta Ha{\bar{\gamma }}_{2}}{\sinh\left(2N\right)}D_{Ha\cdot N}]\cos\left(nx\right)$$

(A20)
$$\begin{array}{ll} T_{28}= & [\frac{\xi_{2}\beta {\bar{A}}_{2}M}{\sinh\left(2N\right)}H_{M\cdot N}+\frac{\xi_{2}\beta {\bar{\gamma }}_{5}\lambda_{21}}{\sinh\left(2N\right)}E_{\lambda_{21}\cdot N}+\frac{\xi_{2}\beta {\bar{\gamma }}_{6}\lambda_{22}}{\sinh\left(2N\right)}E_{\lambda_{22}\cdot N}+\frac{\xi_{2}\beta {\bar{\gamma }}_{7}\lambda_{23}}{\sinh\left(2N\right)}E_{\lambda_{23}\cdot N}\\ & +\frac{\xi_{2}\beta {\bar{\gamma }}_{8}\lambda_{24}}{\sinh\left(2N\right)}E_{\lambda_{24}\cdot N}]\cos\left(nx\right)\cos\left(mx\right) \end{array}$$

(A21)
$$\begin{array}{ll} T_{29}= & [-\frac{\xi_{2}\beta {\bar{A}}_{3}N}{\sinh\left(2N\right)}B_{N}+\frac{\xi_{2}\beta {\bar{\gamma }}_{9}\lambda_{31}}{\sinh\left(2N\right)}E_{\lambda_{31}\cdot N}+\frac{\xi_{2}\beta {\bar{\gamma }}_{10}\lambda_{32}}{\sinh\left(2N\right)}E_{\lambda_{32}\cdot N}+\frac{\xi_{2}\beta {\bar{\gamma }}_{11}\lambda_{33}}{\sinh\left(2N\right)}E_{\lambda_{33}\cdot N}\\ & +\frac{\xi_{2}\beta {\bar{\gamma }}_{12}\lambda_{34}}{\sinh\left(2N\right)}E_{\lambda_{34}\cdot N}]\cos^{2}\left(nx\right) \end{array}$$

(A22)
$$B_{i}=-1+\frac{\sinh\left(4i\right)}{4i}$$

(A23)
$$C_{i}=\cosh\left(2i\right)-\frac{\cosh\left(i\right)\sinh\left(i\right)}{i}$$

(A24)
$$D_{i\cdot j}=\frac{\exp\left(-i\right)j-\exp\left(i\right)j\cosh\left(2j\right)+\exp\left(i\right)i\sinh\left(2j\right)}{i^{2}-j^{2}}$$

(A25)
$$E_{i\cdot j}=\frac{\exp\left(-i\right)\left[\exp\left(2i\right)j-j\cosh(2j)-i\sinh\left(2j\right)\right]}{i^{2}-j^{2}}$$

(A26)
$$F_{i\cdot j\cdot k\cdot l}=\left(\xi_{i}{\bar{\gamma }}_{j}-\xi_{k}{\bar{\gamma }}_{l}\right)$$

(A27)
$$G_{i\cdot j}=-\frac{\sinh\left[2\left(i-j\right)\right]}{2\left(i-j\right)}+\frac{\sinh\left[2\left(i+j\right)\right]}{2\left(i+j\right)}$$

(A28)
$$H_{i\cdot j}=\frac{j\sinh\left(2i\right)-i\sinh\left(2j\right)}{i^{2}-j^{2}}$$

(A29)
$$\left({\bar{A}}_{11},{\bar{A}}_{12},{\bar{A}}_{2},{\bar{A}}_{3}\right)=\frac{\left(A_{11},A_{12},A_{2},A_{3}\right)}{E_{s}u_{r}}$$

(A30)
$${\bar{\gamma }}_{i}=\frac{\gamma_{i}}{E_{s}u_{r}}\;,\;i=1,2,5,6,7,8,9,10,11,12$$
